# Antibody V_h_ Repertoire Differences between Resolving and Chronically Evolving Hepatitis C Virus Infections

**DOI:** 10.1371/journal.pone.0025606

**Published:** 2011-09-28

**Authors:** Vito Racanelli, Claudia Brunetti, Valli De Re, Laura Caggiari, Mariangela De Zorzi, Patrizia Leone, Federico Perosa, Angelo Vacca, Franco Dammacco

**Affiliations:** 1 Department of Internal Medicine and Clinical Oncology, University of Bari Medical School, Bari, Italy; 2 Experimental and Clinical Pharmacology Unit, Centro di Riferimento Oncologico, IRCCS National Cancer Institute, Aviano (PN), Italy; University of Cape Town, South Africa

## Abstract

Despite the production of neutralizing antibodies to hepatitis C virus (HCV), many patients fail to clear the virus and instead develop chronic infection and long-term complications. To understand how HCV infection perturbs the antibody repertoire and to identify molecular features of antibody genes associated with either viral clearance or chronic infection, we sequenced the V(D)J region of naïve and memory B cells of 6 persons who spontaneously resolved an HCV infection (SR), 9 patients with a newly diagnosed chronically evolving infection (CE), and 7 healthy donors. In both naïve and memory B cells, the frequency of use of particular antibody gene subfamilies and segments varied among the three clinical groups, especially between SR and CE. Compared to CE, SR antibody genes used fewer VH, D and JH gene segments in naïve B cells and fewer VH segments in memory B cells. SR and CE groups significantly differed in the frequency of use of 7 gene segments in naïve B cell clones and 3 gene segments in memory clones. The nucleotide mutation rates were similar among groups, but the pattern of replacement and silent mutations in memory B cell clones indicated greater antigen selection in SR than CE. Greater clonal evolution of SR than CE memory B cells was revealed by analysis of phylogenetic trees and CDR3 lengths. Pauciclonality of the peripheral memory B cell population is a distinguishing feature of persons who spontaneously resolved an HCV infection. This finding, previously considered characteristic only of patients with HCV-associated lymphoproliferative disorders, suggests that the B cell clones potentially involved in clearance of the virus may also be those susceptible to abnormal expansion.

## Introduction

Deciphering the humoral immune response to hepatitis C virus (HCV) has been challenging. Although virus-specific antibodies are produced in essentially all persons infected with HCV, about 80% of these patients develop persistent infection and are at risk of long-term complications [1], [2]. The most prevalent of these complications are liver cirrhosis and hepatocellular carcinoma [3], but HCV-infected persons may also develop mixed cryoglobulinemia (MC) and B cell non-Hodgkin lymphoma (B-NHL) [4]–[6]. It is therefore thought that B cells are largely ineffective in resolving HCV infection while they are responsible for its lymphoproliferative complications. Greater understanding of the B cell response to HCV may help predict the outcome of the infection in individual patients as well as their risk of developing lymphoproliferative disorders. However, studying the B cell (antibody) response to HCV has been extremely difficult due to the heterogeneous nature of HCV, the lack of a practical and readily available cell culture system to screen antibodies, and the limited resources for studying HCV infection in chimpanzees, the only species susceptible to HCV infection other than humans [7].

At present, knowledge about the B cell response to HCV in humans is limited to two kinds of data. First, it is known that patients' sera contain antibodies that have neutralizing properties in vitro. Such neutralizing antibodies have been found in both self-limiting (i.e. spontaneously resolving) [8] and chronically evolving [9]–[11] HCV infections. Second, there is some information on the repertoire of antibody variable heavy (V_H_) and variable light (V_L_) genes of whole (unfractionated) B cell populations in liver and blood. So far, the antibody repertoire has been analyzed only in chronic infections. In particular, it has been studied in chronically infected patients with lymphoproliferative disorders (MC or B-NHL) for the purpose of detecting subclinical (MC) or frankly malignant (B-NHL) clonal B cell expansions [12]–[20]. There is, however, no knowledge of the antibody repertoire in patients with self-limiting HCV infection and, importantly, no published study has reported on the antibody repertoire in the two distinct B cell subsets: naïve and memory.

Diversity in the repertoire of antibody H chains is mainly achieved during normal B cell ontogeny (maturation) by random recombination of V_H_, D, and J_H_ segments and by enzymatic modification (addition or deletion of short coding sequences at the VD and DJ joints) of the V_H_DJ_H_ junctions [21]. Single V_H_, D and J_H_ genes are chosen from a repertoire consisting of approximately 40 functional V_H_ gene segments (that are grouped into 7 structurally related families on the basis of at least 80% nucleotide sequence identity), 25 D segments and 6 J_H_ segments. An additional process of sequence diversification is achieved by somatic hypermutation after ontogeny, when mature naïve B cells encounter antigens, undergo rapid clonal expansion and seed germinal centers, thereby developing into memory B cells that express the distinctive CD27 surface protein [21], [22]. Therefore, somatically mutated variable region genes are the hallmark of memory B cells and their progeny. Although the process of somatic hypermutation has an element of randomness, antigen selection tends to cluster silent (S) mutations in the antibody framework regions (FRs), which are required to maintain structural integrity, while replacement (R) mutations are more often found in the complementarity-determining regions (CDRs), which form the antigen binding sites [21], [23]–[25].

The H chain CDR 3 (CDR3_H_), located at the junction of the V_H_, D, and J_H_ segments, is the most diverse region in the antibody molecule. For this reason, it is considered to represent a molecular footprint of the overall antibody repertoire. Structurally, the CDR3_H_ is located in the center of the antigen-binding site and interacts directly with other CDRs and FRs from both H and L chains, as well as with the antigen itself [26]. Its length varies in a Gaussian-like distribution in physiological conditions, while alterations away from this normal profile suggest B cell selection and clonal expansion [27], [28]. Changes in the length and amino acid composition of CDR3_H_ directly affect the charge, hydrophobicity, size and shape of the antigen-binding site [29] and, thus, the ability of the antibody to bind antigen. The present study was therefore conducted to investigate potential differences in the antibody repertoire of persons who spontaneously resolved HCV infection and from subjects who became chronically infected with HCV. For this purpose, we cloned and sequenced the DNA of the V(D)J region of naïve and memory B cell fractions and determined the frequency of usage of individual V_H_, D and J_H_ families and subfamilies in these two clinical populations and in healthy persons.

## Results

To understand if and how HCV infection perturbs B cell antigen receptor (antibody) repertoire and how this is associated with the outcome of HCV infection, we cloned and sequenced the V(D)J region of circulating CD27^−^ (naïve) and CD27^+^ (memory) B cells from 7 healthy donors (HD), 6 persons who spontaneously resolved (SR) an HCV infection, and 9 patients with chronically evolving (CE) HCV infection (Table 1).

**Table 1:** Demographic, clinical, and virological parameters for 6 Caucasian persons who spontaneously resolved (SR) an HCV infection and 9 Caucasian patients with a recently diagnosed chronically evolving (CE) HCV infection.

Subject	Age(yr)	Sex	HCVGenotype	ALT(U/l)	HCV RNA(IU/ml)	Anti-HCVAbs
			(**cleared virus**)			
SR1	48	F	1b	25	-	+
SR2	61	F	2a	15	-	+
SR3	35	M	2a	37	-	+
SR4	50	M	1b	28	-	+
SR5	65	M	2a/2c	41	-	+
SR6	58	F	1a	39	-	+
			(**persistent virus**)			
CE1	47	F	1b	67	17,902	+
CE2	29	F	2a/2c	58	297,411	+
CE3	35	F	1b	74	56,693	+
CE4	65	M	1b	84	1,930,060	+
CE5	50	M	2a	55	405,357	+
CE6	56	F	1b	211	2,388,080	+
CE7	59	M	1a	95	985,380	+
CE8	48	M	1b	45	373,745	+
CE9	52	F	1a	65	227,320	+

*yr*, years; *F*, female; *M*, male; *ALT*, alanine aminotransferase; *Abs*, antibodies.

**Figure 1:**
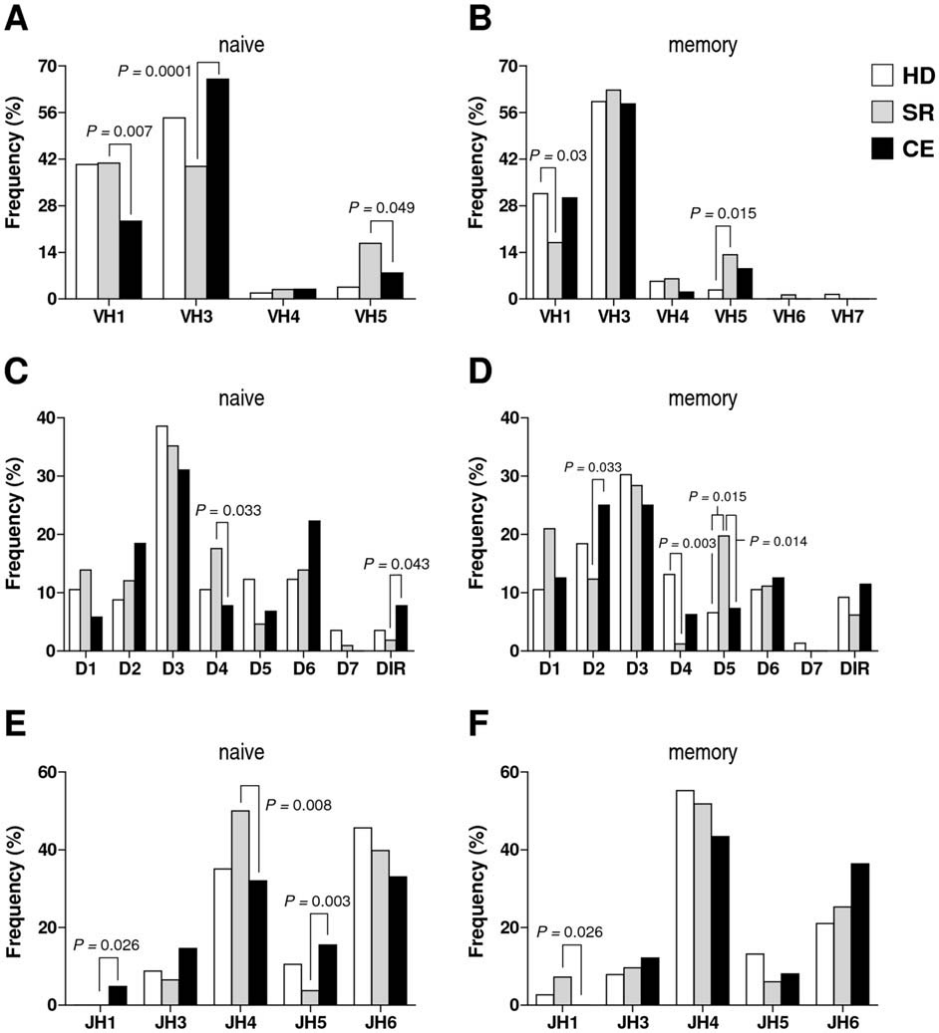
Frequency of usage of V(D)J subfamilies in molecular clones of naïve and memory B cells from 7 healthy donors (HD), 6 persons who spontaneously resolved an HCV infection (SR) and 9 patients with chronically evolving HCV infection (CE). For each subject, 32 clones from naïve B cells and another 32 from memory B cells were analyzed. Values represent the percentage of subfamily use for all clones in a particular clinical group and B cell subset. *Panels* ***A*** *,* ***C*** *and* ***E***, naïve B cell clones; *panels* ***B*** *,* ***D*** *and* ***F***, memory B cell clones. Associations between clinical group and individual subfamily were tested for significance with chi-square or Fisher's exact test only when a previous chi-square test indicated a significant association of gene family usage in a pairwise comparison of groups; p values are shown only when ≤0.05.

First, we determined the frequency of usage of individual V_H_, D and J_H_ subfamilies in the three clinical groups (Figure 1). In naïve B cell clones, the most abundant V_H_ subfamilies in all three groups were V_H_1 and V_H_3 and, of the other V_H_ subfamilies, only V_H_4 and V_H_5 were also found (Figure 1A). The overall pattern of V_H_ usage was significantly associated with clinical group in the pairwise comparison between SR and CE (p = 0.002, chi-square test); at the subfamily level, these two patient groups gave significant associations in the use of V_H_1, V_H_3 and V_H_5. In contrast, no significant association in V_H_ usage was found in comparisons of HD to either SR (p = 0.06) or CE (p = 0.13). In memory B cell clones (Figure 1B), the pattern of prevalent V_H_1 and V_H_3, followed by V_H_5, was maintained although a small percentage of clones also used V_H_6 and V_H_7; statistical significance was observed for the comparison between HD and SR (p = 0.027, attributable to V_H_1 and V_H_5), but not for HD vs. CE (p = 0.24) or SR vs. CE (p = 0.11). For the D gene family (Figure 1C, D), both naïve and memory B cell clones used D1–D7 as well as DIR, and the D3 subfamily was predominant. In naïve clones (Figure 1C), the pattern of usage was significantly associated with clinical group for the comparison SR vs. CE (p = 0.021, attributable to D4 and DIR) but not between HD and either SR (p = 0.43) or CE (p = 0.09). In memory clones (Figure 1D), the usage patterns were significantly associated with group in comparisons between HD and SR (p = 0.009, attributable to D4 and D5) and between SR and CE (p = 0.018, attributable to D2 and D5), but not between HD and CE (p = 0.68). Finally, of the six J_H_ subfamilies, all were used to some extent except J_H_2; subfamilies J_H_4 and J_H_6 were prominent in both naïve and memory clones (Figure 1E, F). In naïve clones (Figure 1E), usage patterns were significantly associated with group in the comparison between SR and CE (p = 0.003, attributable to J_H_1, J_H_4 and J_H_5) but not between HD and either SR or CE (p = 0.26 and p = 0.19, respectively). Similarly, in memory clones (Figure 1F), a significant association was found in the comparison of the two patient groups SR and CE (p = 0.039, attributable to J_H_1) but not in comparisons between HD and either SR or CE (p = 0.36 and p = 0.06, respectively). This analysis suggests that, despite general similarities in the use of V_H_, D and J_H_ subfamilies between naïve and memory B cell clones, the patterns of subfamily usage in the three clinical groups differ, especially between SR and CE, and that within these groups the proportions of individual subfamilies in naïve B cells do not always match the proportions of the corresponding subfamilies in memory B cells.

**Figure 2:**
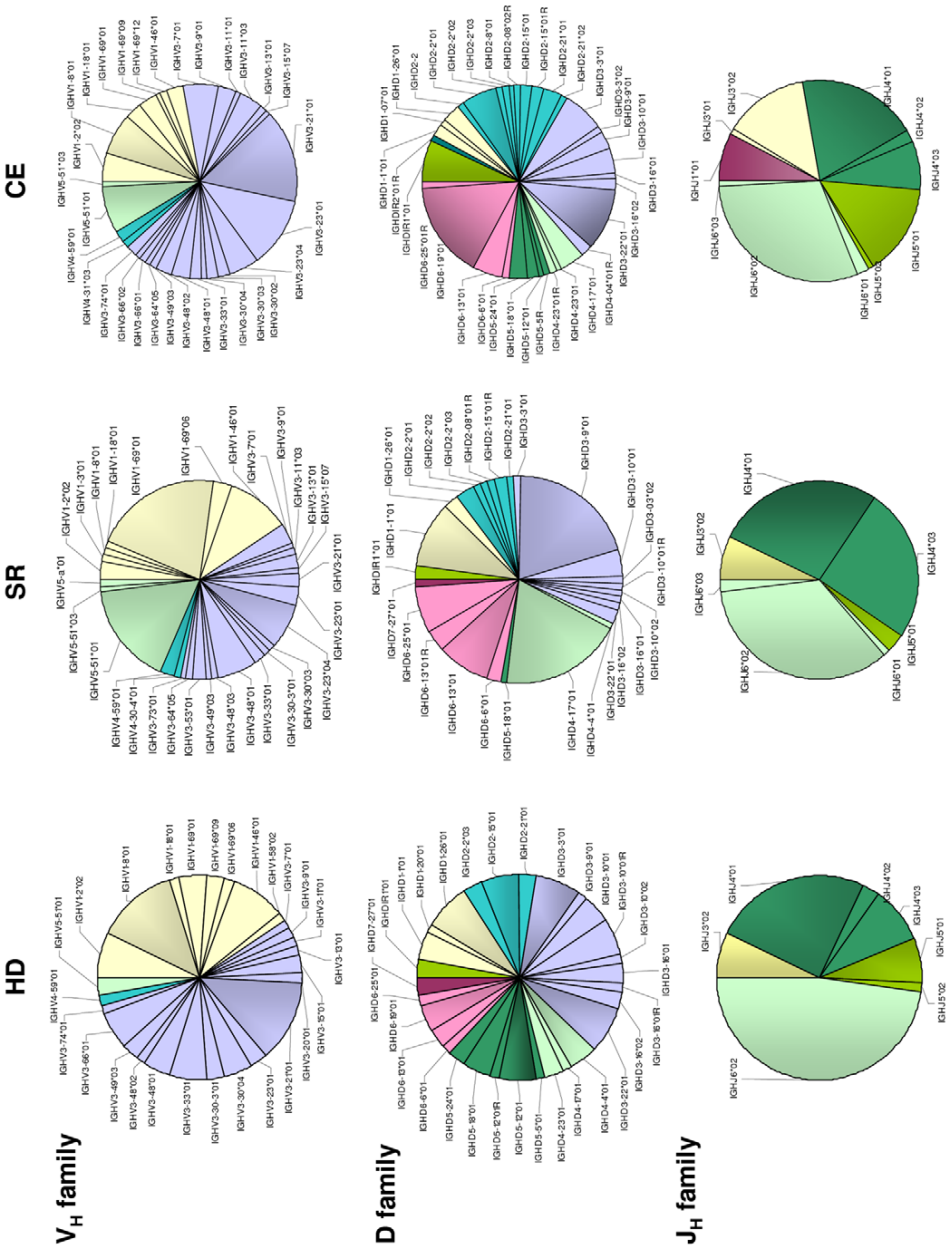
Naïve B cells: fractional usage of individual V(D)J gene segments in molecular clones from healthy donors (HD), persons who spontaneously resolved an HCV infection (SR) and patients with chronically evolving HCV infection (CE).

We next identified the specific gene segments used by the three clinical groups, beginning with the subset of 704 clones representing naïve B cells (Figure 2). Overall, we identified 26 different V_H_ family gene segments in HD, 29 in SR, and 31 in CE; 13 of these gene segments were used by all three groups. The numbers of D family gene segments found in HD, SR and CE clones were 29, 26 and 34, respectively, and 15 gene segments were in common. Finally, we identified 7, 7 and 11 different J_H_ family gene segments in the same clinical groups (5 in common). This analysis suggests that a wider variety of gene segments is used in naïve B cells from CE samples compared to both HD and SR.

**Figure 3:**
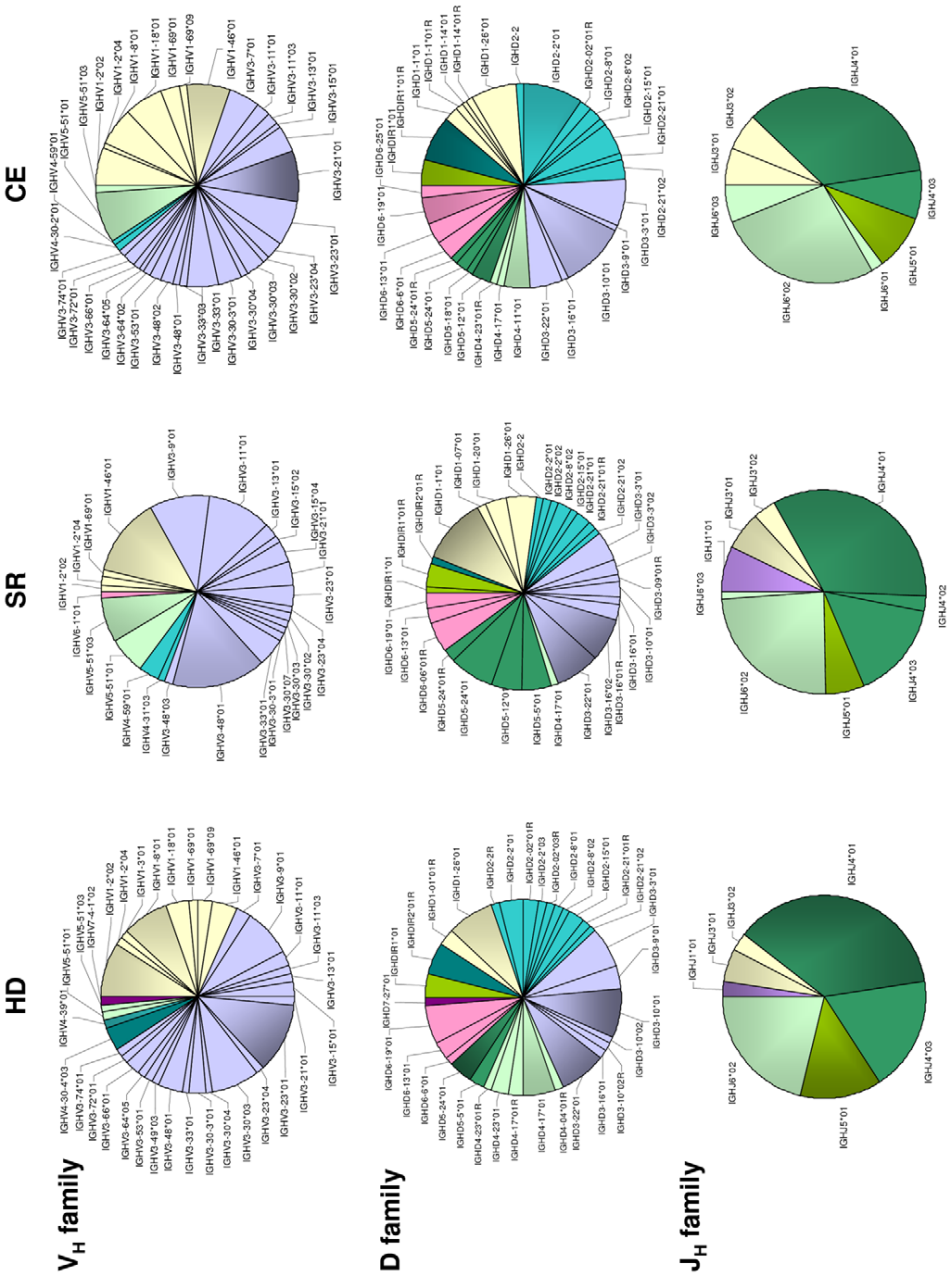
Memory B cells: fractional usage of individual V(D)J gene segments in molecular clones from healthy donors (HD), persons who spontaneously resolved an HCV infection (SR) and patients with chronically evolving HCV infection (CE).

The same analysis was performed for the 704 clones representing memory B cells (Figure 3). Here, we identified 33 different V_H_ family gene segments in HD, 24 in SR, and 34 in CE; 15 gene segments were in common for all three groups. The numbers of D family gene segments found in HD, SR and CE clones were 32, 31 and 31, respectively; 14 gene segments were in common. Finally, we identified 7, 9 and 8 different J_H_ family gene segments in the same clinical groups (6 in common). The tendency for a wider variety of gene segments in CE samples, already observed in clones from naïve B cells, was maintained for the V_H_ family versus SR only, but not for the D or J_H_ family.

Then, the frequency of usage of individual gene segments in clones from naïve and memory B cells was analyzed statistically for those subfamilies that gave significant associations with clinical group (as indicated in Fig. 1). Despite the fact that numerous gene segments were used in common by the clinical groups, only a limited number of alleles was significantly associated with group (Table 2). In the analysis of naïve B cell clones (where significance was found only between SR and CE), this included one V_H_1 gene segment, namely IGHV1-69*01, as well as two V_H_3 gene segments and one each from the D4, J_H_1, J_H_4 and J_H_5 subfamilies; no significant association was found for V_H_5 or DIR. In the analysis of memory B cell clones, three V_H_1 gene segments were significantly associated with clinical group in the comparison of HD and SR and, comparing SR and CE, a significant association was found for one gene segment each in subfamilies D2, D5 and J_H_1. Altogether, this analysis shows that B cells from the three clinical groups differ in the frequency of usage of particular V(D)J gene segments.

**Table 2:** Frequency of usage of V(D)J gene segments in 1408 molecular clones from 7 healthy donors (HD), 6 persons who spontaneously resolved (SR) an HCV infection and 9 patients with chronically evolving (CE) HCV infection, by B cell subset.

Gene segment	Frequency, %			p value	
	HD	SR	CE	HD vs. SR	SR vs. CE
Naïve B cells					
IGHV1-69*01	7.0	18.5	2.9	ND	<0.001
IGHV3-21*01	12.3	3.7	15.5	ND	0.004
IGHV3-23*01	3.5	2.8	11.7	ND	0.015
IGHD4-17*01	1.8	16.7	3.9	ND	0.003
IGHJ1*01	0.0	0.0	4.9	ND	0.026
IGHJ4*03	10.5	23.1	7.8	ND	0.002
IGHJ5*01	8.8	3.7	14.6	ND	0.007
Memory B cells					
IGHV1-2*02	9.2	1.2	6.1	0.028	ND
IGHV1-8*01	7.9	0.0	6.1	0.011	ND
IGHV1-46*01	3.9	13.3	7.1	0.050	ND
IGHD2-2*01	3.9	1.2	9.4	ND	0.022
IGHD5-5*01	2.6	4.9	0.0	ND	0.042
IGHJ1*01	2.6	7.2	0.0	ND	0.008

Chi-square or Fisher's exact test was performed for individual gene segments only when the pairwise comparison of two clinical groups gave a significant association at the subfamily level; data are shown only when p≤0.05.

*ND*, not determined (no significant association at gene subfamily level).

Each V_H_ gene segment was then scrutinized for somatic mutations with respect to the germline gene with highest sequence similarity (Table 3). An unmutated germline sequence was found in a small percentage of clones from naïve B cells from all three clinical groups, whereas clones from memory B cells all contained mutations. Naïve B cell clones from HD tended to have a low rate of mutation, with over 60% of clones being categorized in the ≤2% mutated nucleotide class; in SR and CE, the percentage of clones in this low mutation category were 36.5% and 47.6%. Memory B cell clones had a high rate of mutation, with 93.4% of all HD clones being categorized in the >2% nucleotide mutation class; in SR and CE, these values were lower (84.3% and 75.8%, respectively). These findings suggest that B cells recently exposed to HCV, irrespective of infection outcome, have a general perturbation in their mutation rate.

**Table 3:** Percentages of B cell molecular clones with somatic mutations in V_H_ gene, by B cell fraction and clinical group.

Mutation frequency	Naive B cell clones, %			Memory B cell clones, %		
	HD	SR	CE	HD	SR	CE
No mutations	2.9	3.1	1.9	0	0	0
≤2% of all nucleotides	60.9	36.5	47.6	6.6	15.7	24.2
>2% of all nucleotides	36.2	60.4	50.5	93.4	84.3	75.8

**Figure 4:**
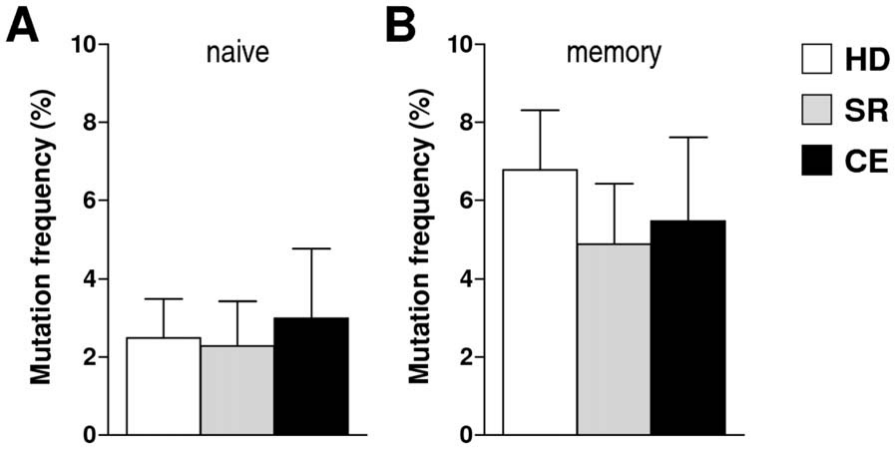
Mutation frequencies of V_H_ gene segments in molecular clones of naïve B cells (A) and memory B cells (B), by clinical group. Values are mean (SD) of the per-subject average (32 clones per subject). p<0.001, all naïve vs. all memory clones, chi-squared test.

The V_H_ gene mutation frequency was then determined for naïve and memory B cell clones from each individual and averaged per clinical group (Figure 4). The mutation frequency was low (2%–3%) for naïve B cell clones, without a significant association with clinical group (one-way ANOVA, p = 0.61) (Fig. 4A). For memory B cell clones, these values were about two-fold higher, again without a significant association with group (p = 0.17) (Fig. 4B). However, the mutation frequency of V_H_ gene sequences from all memory B cell clones together was significantly higher than that of naïve B cell clones (p<0.001, chi-squared test).

**Figure 5:**
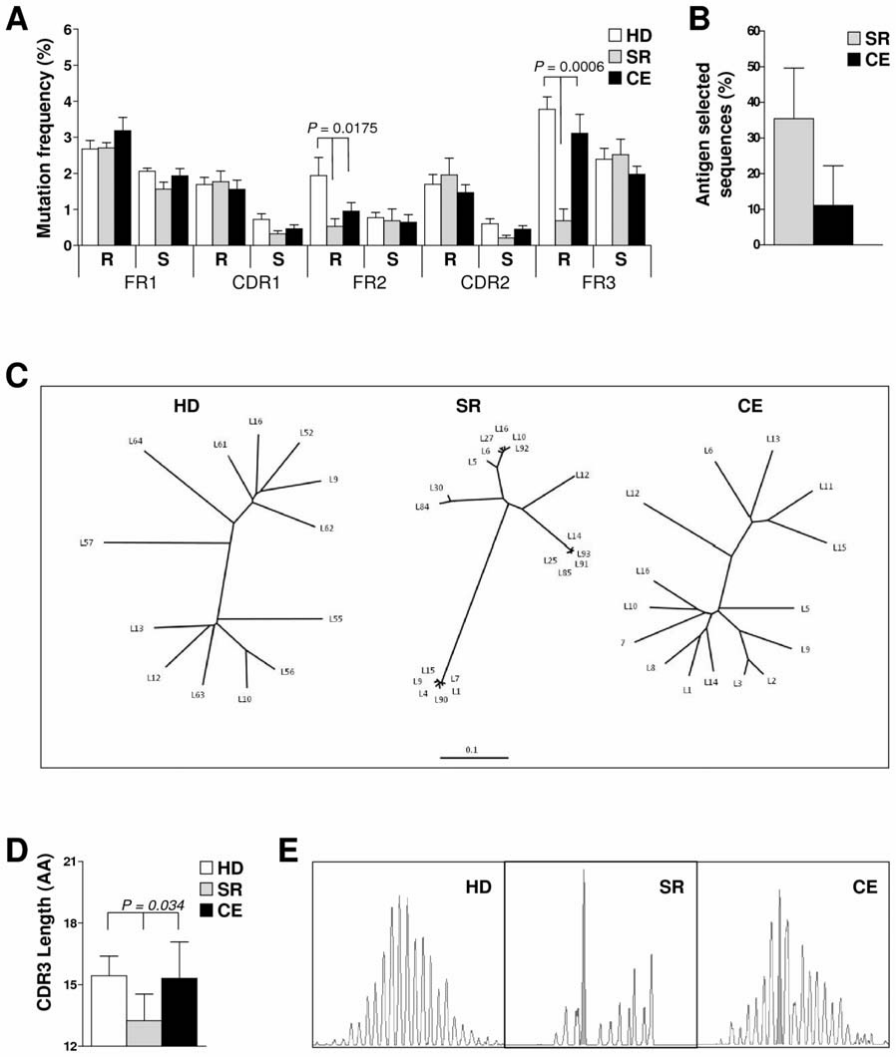
Evidence for antigen selection of V_H_ gene segments in molecular clones from memory B cells. A Distribution of replacement (R) and silent (S) mutations in each framework region (FR) and complementarity-determining region (CDR), by clinical group. Statistical significance by ANOVA is indicated. B Percentage of V_H_ gene segments with evidence of antigen selection, according to Lossos et al. [45]. Values are mean (SD) of the per-subject average (32 clones per subject). C Representative phylogenetic trees from one healthy donor (HD), one patient who spontaneously resolved an HCV infection (SR), and one patient with chronically evolving HCV infection (CE). The pattern of branching and clustering of sequences in the SR tree is evidence of clonal evolution. D Length of CDR3 peptide loops deduced from V_H_ gene nucleotide sequences of memory B cells, by clinical group. Values are mean (SD) of the average value per subject. ANOVA p = 0.034; Newman-Keuls post test: HD vs. SR, p = 0.0.017; HD vs. CE, p = 1.0; SR vs. CE, p = 0.042. E Representative CDR3 length distribution profiles determined by spectratyping on CDR3 PCR products reveal evidence of clonal selection in SR.

Given the higher mutation frequency in memory B cell clones, these genes were further investigated in terms of R and S mutations and their distribution in FR and CDR along the V_H_ gene (Figure 5A, B). A significant association between R mutation frequency and clinical group was observed in FR2 and FR3, with the highest value in HD and the lowest value in SR. In CDR1 and CDR2, the frequencies of R and S mutations were similar among groups, yet SR had the highest frequency of R mutations and the lowest frequency of S mutations in both regions; this resulted in SR having a higher R∶S ratio for CDR (14.8) than either HD (3.5) or CE (4.4), suggesting antigen selection (Figure 5A). To further understand if the somatic mutation pattern in memory B cell clones was characteristic of antigen selection, each V_H_ gene was analyzed for the probability that the numbers of R mutations in FR and CDR occurred by chance. This probability was assessed using a mathematical algorithm, whereby the expected mutations were compared to the observed mutations, and p values were calculated on the basis of a multinomial distribution. Genes with low p value (<0.05) for both FR and CDR were considered to have undergone antigen selection. This analysis revealed that, on average, 35.4% of V_H_ genes in SR group had undergone antigen selection (SD = 14.6%) compared to 11.1% (SD = 12.6%) in CE (Figure 5B). Altogether, these results suggest that, despite similar V_H_ mutation frequencies in memory clones from the three clinical groups, a distribution of R and S mutations characteristic of antigen selection was more evident in SR than in CE.

To visualize the lineage relationship among antigen-selected sequences, we constructed phylogenetic trees for memory B cell V_H_ sequences from each study subject. In general, in trees from HD and CE, sequences were well distanced from nodes and from each other along long branches, suggesting sequence diversity. By contrast, in SR trees, multiple sequences were clustered on short branches around a few nodes, suggesting clonal evolution (Figure 5C).

To further assess antibody repertoire variations in memory B cell clones, we calculated average CDR3 length per subject and then per study group (Figure 5D). Mean CDR3 length was about 15 residues in HD and CE groups but only about 13 residues in SR (ANOVA p = 0.034, Newmann-Keuls post test, p<0.05 for SR vs. both HD and CE). The shorter CDR3 length in SR suggested a mobilization of the antibody repertoire due to clonal selection. This possibility was confirmed by spectratyping of CDR3 PCR products (Figure 5E), which showed a physiological, Gaussian-like distribution of lengths in HD, a slight deviation from a normal profile in CE, but a complete loss of this pattern with prominent peaks in SR.

**Figure 6:**
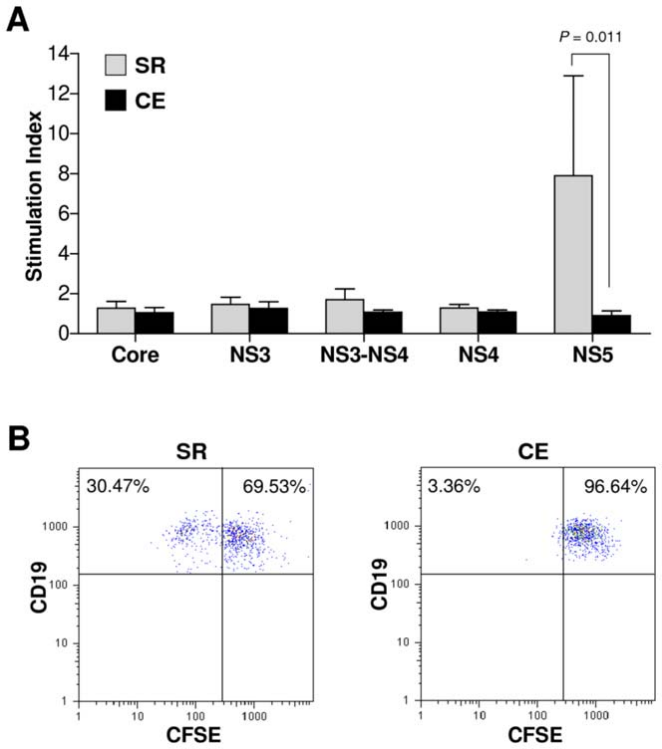
In vitro proliferation of CFSE-labelled B cells in response to 7 days' stimulation with different recombinant HCV-SOD fusion proteins, by clinical group. A Stimulation index, calculated as the percentage of cells with diluted CFSE staining (indicating cell division) after HCV-SOD treatment relative to that of SOD-treated control cells. SR vs. CE, p = 0.011 Mann-Whitney U test after ANOVA. B Representative FACS scatter plots of B cells from SR (*left*) and CE (*right*) stimulated with NS5 HCV-SOD fusion protein show greater CFSE dilution (hence proliferation) in SR.

Finally, to determine if the observed molecular differences between memory B cells from SR and CE had functional correlates, we assessed the ability of freshly purified CD27^+^ B cells to proliferate upon in vitro stimulation with recombinant HCV antigens, by measuring the dilution of the division-tracking dye CFSE after 7 days. For CE samples, mean stimulation index was <2 for all HCV antigens, indicating no proliferative response (Figure 6A). For SR, stimulation index was negative for all antigens but NS5, which produced an abundant proliferative response (Figure 6B). This finding suggests a role of antibody against NS5 in viral clearance.

## Discussion

This study provides a snapshot view of the antibody repertoire of HCV-infected persons shortly after their spontaneous recovery (SR) or in the early period of a chronically evolving infection (CE). In both naïve and memory B cell subsets, the frequency of usage of particular antibody gene subfamilies and segments (alleles) varied in SR, CE and healthy donors (HD), with particular differences between SR and CE. Within these latter two groups, a reversed expression of a few subfamilies distinguished naïve from memory B cells. Compared to CE, the antibody genes of SR were composed of fewer V_H_, D and J_H_ gene segments in naïve B cells and fewer V_H_ segments in memory cells. Although the nucleotide mutation rate was similar among clinical groups, the pattern of replacement and silent mutations in memory B cell clones gave evidence for greater antigen selection in SR than CE samples. Greater clonal evolution of SR than CE memory B cells was also supported by analysis of phylogenetic trees and CDR3 lengths. Finally, freshly purified B cells from SR but not CE gave a proliferative response to in vitro stimulation with NS5 HCV-SOD fusion protein.

The most intriguing finding of this study is that antibody V(D)J sequences are encoded by a smaller number of germline elements in naïve and memory B cells from persons who spontaneously resolved an HCV infection than from patients with a chronically evolving infection. Until now, a restricted repertoire of antibody variable heavy (V_H_) genes and a clustering of V_H_ gene somatic mutations have been regarded as hallmarks of HCV-associated MC and B-NHL and have been interpreted as direct consequences of persistent antigenic stimulation of B cells [12]–[17], [19], [30]. We instead found that these features are common to self-limiting infections: this suggests that B cell clones that are potentially involved in clearance of the virus are also those susceptible to abnormal clonal expansion. This speculation is consistent with a recent study by Charles et al. [31], who found that some patients with HCV-associated MC expressed a common subset of clonally expanded, weakly hypermutated, antibody gene segment-restricted, memory-like B cells.

Particularly puzzling are the differences of the V(D)J repertoire between naïve and memory B cells of the CE group. For example, we observed that some genes were relatively enriched in memory B cells but depleted in naïve B cells; this difference could be due to the transfer of some B cells from the CD27^−^ to the CD27^+^ subset because of their memory differentiation. Other genes were characterized by a relative enrichment in naïve B cells but a depletion in memory B cells; this pattern may be due to changes in B cell selection or to the presence within the CD27^−^ fraction of atypical memory B cells that have undergone isotype switching and somatic hypermutation, but have downregulated CD27. Our finding of the highest percentage of naïve B cell clones with >2% mutated nucleotides in the CE group supports this possibility. These atypical memory B cells have already been described by our group [32] and have recently been detected in HIV-infected [33] and *Plasmodium falciparum*-infected [34], [35] individuals. They have been named ‘exhausted’ memory B cells because of their increased expression of inhibitory receptors, altered expression of homing receptors, reduced proliferative potential and stunted replication history and immunoglobulin diversity [36].

The study also reveals that the antibody V(D)J sequences of naïve B cells from both SR and CE lack molecular signs (e.g. high mutation rate) of current activation. This result contrasts with the idea that HCV-associated abnormal clonal B cell populations arise from naïve B cells and does not confirm the phenotypic findings of high levels of activated naïve B cells in the blood of HCV^+^ patients with MC [37]. The finding that B cells from SR proliferated upon stimulation with a nonstructural HCV antigen (NS5) proves that these cells are specific for this protein. Whether this specificity contributes to infection control because it is expressed by direct antibody generation or, alternatively, by CD4 T cell help remains to be determined. Indeed, our understanding of the direct role of antibodies in viral protection and disease outcome emerges from numerous clinical and experimental observations, as summarized here. Patients suffering from agammaglobulinemia experience an accelerated disease progression [38]. Immunoglobulin produced from pooled human plasma negative for anti-HCV antibodies transmitted hepatitis C to chimpanzee recipients, while that from unscreened pooled plasma was found to contain neutralizing antibodies that prevented infection [39]. Polyclonal antibodies isolated from a chronic HCV-infected patient protected chimeric mice against in vivo challenge with different HCV genotypes, although the efficacy of such cross-genotype neutralization was lower than that predicted by cell culture experiments [40]. In HCV-infected liver transplant recipients, the HCV variants that infected the liver grafts were poorly neutralized by antibodies present in the patients' pre-transplant sera (rather than variants no longer detected after transplantation) [41]. Finally, while in self-limiting infections neutralizing antibodies can be rapidly detected in the serum and progressively decrease or even disappear after recovery, in chronically evolving infections they appear later and tend to persist [8], [42].

There are some caveats associated with the current study. First, memory B cells used in the experiments reported here were not selected for antigen specificity, due to the well-known technical difficulties of achieving pure cell populations. This may have biased our results given that it is unclear how representative the overall B cell repertoire (which is not shaped only by HCV) is of the HCV-specific B cell compartment. On the other hand, by using whole B cells, we did not exclude from study those cells that are involved in the immune pathogenesis of HCV infection but that do not directly bind the viral particle. This permitted us to get a more complete picture of the B cell response to HCV. Second, some sequences might have been cloned more readily than others after PCR amplification. To minimize the risk of frequency underestimation, we extended the number of clones up to 32 per B cell subset per patient: as already demonstrated, when an adequate number of templates is amplified, results of PCR-cloning do not significantly differ from those obtained by single genome analysis [43]. In addition, we used degenerated primers to amplify all V_H_ families and intentionally omitted pre-PCR sampling (i.e. single-cell sorting) to avoid potential underestimation of the genetic diversity of the antibody sequences. Another limitation of the study is the small number of subjects studied, due to the fact that conducting such an extensive and articulated work is difficult in a larger sample. Therefore, these findings should be confirmed by further studies in other larger and more diverse HCV-positive populations (i.e. patients before and after complete viral eradication, anti-HCV therapy responders vs. non-responders, persons with different IL-28B genotypes).

In conclusion, this study found that pauciclonality of the memory B cell population is characteristic of persons who had a recent, self-limiting HCV infection. This observation leads us to hypothesize a model of pathogenesis in which the antibody response to HCV may follow three different pathways: 1) selected B cell subsets are activated, undergo memory differentiation and clonal expansion, produce hypermutated and affinity-matured antibodies and then contract when viral clearance is achieved (resolved infection); 2) B cell subsets are activated as in scenario 1 but continue to abnormally expand if antigen persists (chronic infection complicated by MC); and 3) B cells are not efficiently activated and produce low-affinity antibodies without protective ability (chronic infection without MC). It is currently unknown why, in some patients, an initially successful B cell response deviates towards a proliferative disorder (scenario 2), but both viral and host factors may be involved. Whether our data might be used to inform therapeutic interventions or vaccine development in terms of antigen design or dosing remains to be assessed. Future studies should indeed compare SR and CE groups for differences in B cell epitopes of the virus, HLA polymorphisms, and B-T cell cognate interactions.

## Materials and Methods

### Study subjects and biological samples

Peripheral blood samples were obtained from 7 healthy donors (HD), 6 patients who had spontaneously resolved an acute HCV infection in the 6 months prior to sampling (spontaneous resolvers, SR), and 9 patients with a recent (<9 months) diagnosis of chronic HCV infection (chronically evolving, CE), defined as an infection of at least 6 months' duration. All subjects were Caucasian, negative for antibodies to human immunodeficiency virus, antibodies to hepatitis B virus, hepatitis B surface antigen, serum rheumatoid factor and cryoglobulins. None had ever had antiviral therapy nor a diagnosis of lymphoproliferative disorders.

The study protocol was approved by the University of Bari Medical School Ethics Committee and conformed to the good clinical practice guidelines of the Italian Ministry of Health and the ethical guidelines of the Declaration of Helsinki, as revised and amended in 2008. Written informed consent was obtained from each subject.

### Cell preparations

Peripheral blood mononuclear cells (PBMC) were separated by Ficoll-Hypaque (Pharmacia Biotec, Uppsala, Sweden) density gradient centrifugation. B cells were isolated from PBMC by automated magnetic cell sorting using the B Cell Isolation Kit II (Miltenyi Biotec). B cells were further fractionated into naïve (CD27^−^) and memory (CD27^+^) populations by automated magnetic cell sorting with anti-CD27 microbeads (Miltenyi Biotec). All sorted cell populations exhibited >95% purity, as revealed by staining with either peridin chlorophyll protein-conjugated anti-CD19 or fluorescein isothiocyanate-conjugated anti-CD27, followed by analysis with a FACSCanto (Becton Dickinson) cytometer and FlowJo software (Tree Star) (Figure S1). Isolated cells were cryopreserved until use.

### Amplification, cloning and sequencing of the V(D)J gene region

Genomic DNA was extracted from naïve and memory B cell fractions using the BioRobot EZ1 Workstation and the EZ1 DNA Tissue kit (Qiagen). The V(D)J region was amplified with a semi-nested protocol according to an established procedure [13]. Briefly, the upstream primer was complementary to the first framework V (variable) region (FR1) [44] and the downstream primer annealed to an outer conserved sequence of the joining region (J_H_) in the first round and to an inner conserved sequence of the same J_H_ in the second round of amplification. PCR products were resolved on 1.3% agarose gels stained with ethidium bromide, and optically analyzed by ultraviolet transillumination. The bands comprised between 350 and 400 bp were excised and purified using the Montage DNA Gel Extraction Kit (Genomixs). PCR products were cloned using the TOPO TA cloning system (Invitrogen Life Technologies). Randomly selected bacterial colonies were picked, cultured and extracted for plasmid DNA using QIAprep Miniprep Kit (Qiagen). DNA was sequenced on the ABI 3100 Genetic Analyzer (Applied Biosystems) using the BigDye Terminator v3.1 Cycle Sequencing Kit (Applied Biosystems) [30]. For each subject, at least 32 clones were sequenced per sample (naïve and memory B cells).

### Analysis of V(D)J gene sequences

Results from automated DNA sequencing were inspected and validated using Chromas Lite 2.01 software (Technelysium, Tewantin, Australia). Only productive rearranged sequences (no stop codons or in frame junctions) were further analyzed. For each subject, 704 sequences were analyzed for naïve B cells and another 704 sequences for memory B cells, for a total of 1408 sequences. The variable (V), diversity (D) and joining (J) genes and alleles were identified by comparison with germline antibody genes, using the International ImMunoGeneTics (IMGT) information system (http://imgt.cines.fr) and the NIH Joinsolver (http://joinsolver.niams.nih.gov/index.htm) with default settings. For each gene, alleles were grouped by subfamily and expressed as a percentage in naïve and memory subsets of each clinical group.

Using the same software, the percentage of mutated nucleotides per V_H_ segment was determined and used to categorize naïve and memory clones into three groups: no mutations, ≤2% mutated nucleotides or >2% mutated nucleotides as suggested by Lossos et al. [45] Then, for each subject, the frequency of mutated V_H_ nucleotides was determined as the number of mutated nucleotides divided by the total number of nucleotides, from FR1 to FR3 inclusive, for all molecular clones sequenced for the particular person; these values, expressed as a percentage, were used to calculate the mean (SD) mutation frequency for naïve and memory clones for each clinical group. This same calculation was repeated for each FR and CDR within the V_H_ segment for memory B cell clones, distinguishing between replacement (R) and silent (S) mutations as indicated by the IMGT information system.

The antigen selection pressure on Ig genes was calculated according to the multinomial distribution model of Lossos et al. [45], using the online JAVA applet (http://www-stat.stanford.edu/immunoglobulin/). This analysis determines, for each V_H_ gene segment, the probability that a scarcity of R mutations occurred by chance in the CDRs (pCDR), as well as the probability that an excess of R mutations occurred by chance in the FRs (pFR). An antigen-selected sequence was defined as one with both pFR<0.05 and pCDR<0.05. The percentage of antigen-selected sequences per subject was calculated and then averaged per clinical group.

Similarities in V_H_ nucleotide sequences were identified using the multiple sequence alignment application ClustalX2.0.9 [46]. Unrooted phylogenetic trees were constructed with ClustalX2 using distance-based neighbor-joining analysis performed on Tamura-Nei neighbor estimates and visualized with TreeView 1.6.6. [47]. Bootstrap analyses were conducted using 1000 replicates.

### CDR3 analysis

The length of each CDR3 in molecular clones from memory B cells was determined from the deduced protein sequence by counting the number of amino acids between the last residue of FR3 (V_H_) and the first residue of J_H_ (FR4). These values were averaged per subject and used to determine the mean (SD) value for each clinical group.

To obtain a graphical view of CDR3 size distributions, CDR3 DNA from memory B cell clones was amplified using FR3 and J_H_ primers, the PCR product was analyzed on an ABI PRISM 3100 Genetic Analyzer, and spectratyping analysis was performed using ABI Prism 3100 GeneScan 3.7 software, as described [48].

### In vitro proliferation assay

Freshly purified CD27^+^ B cells were labeled with the vital dye 5(6)-carboxyfluorescein diacetate, succinimidyl ester (CFSE; Molecular Probes), as described [32]. Labelled cells were cultured in U-bottom 96-well plates (Falcon, BD Biosciences) in RPMI-1640 medium completed with 10% heat-inactivated fetal bovine serum (FBS), 2 mM L-glutamine, 100 U/ml penicillin, 100 mg/ml streptomycin (all from Sigma-Aldrich). Each well contained 10^5^ cells in 200 µl medium. Wells in triplicate were stimulated with a recombinant fusion protein (10 µg/ml) consisting of superoxide dismutase (SOD) combined with one of five HCV antigens (core, c22, aa 2–120; NS3, c33, aa 1192–1457; NS4, c100, aa 1569–1931; NS3/NS4A, aa 1027–1711; NS5, aa 2054–2995; Chiron). Control wells received 10 µg/ml recombinant SOD or 1 µg/ml phytohemagglutinin. Cells were incubated at 37°C in a humidified atmosphere containing 5% CO_2_ for 7 days, and then analyzed for CFSE fluorescence with a FACSCanto (BD Biosciences) flow cytometer and FACSDiva software (BD Biosciences). The percentage of CD19^+^ cells with diluted CFSE staining (indicating proliferation) was determined and averaged for triplicate samples. The extent of proliferation was determined from the relative loss in fluorescence, by calculating the stimulation index according to the formula: percentage of CFSE-dilute cells in presence of antigen/percentage of CFSE-dilute cells in absence of antigen (recombinant SOD alone). These values were averaged for each antigen and clinical group. A stimulation index 3 standard deviations higher than the average stimulation index of healthy donor cells was taken to indicate a proliferative response to antigen.

### Statistical analysis

Statistical analyses were performed using Statistica 6.1 software (StatSoft) and assumed independence between cell clones, i.e. ignored any possible correlation between data obtained from different cells from the same individual.

The patterns (frequencies) of V_H_, D and J_H_ gene family use were tested for significance with a step-by-step procedure. First, the chi-square test was performed in pairwise comparisons of clinical groups, separately for naïve and memory B cell subsets. When this test indicated a significant association at the gene family level, further statistical analyses using chi-square or, when necessary, Fisher's exact test were performed for gene subfamilies. Then, for those subfamilies whose usage was significantly associated with clinical group, chi-square or Fisher's exact test was used again for individual genes.

Mean percentages of nucleotide mutations and mean CDR3 lengths were compared among clinical groups by one-way ANOVA with Newman-Keuls post test. Mean percentages of mutations in naïve and memory B cells were compared using chi-squared test. Stimulation indexes were compared by one-way ANOVA with Mann-Whitney U post test. Values of p≤0.05 were assumed to be significant.

## Supporting Information

Figure S1 Representative immunomagnetic purification of CD27^+^ and CD27^−^ B cells.
(TIF)
