# Supplementary figures and images for: Antibody Vh Repertoire Differences between Resolving and Chronically Evolving Hepatitis C Virus Infections
Source: PLoS One. 2011 Sep 28;6(9):e25606. doi: 10.1371/journal.pone.0025606 (PMC3182224; doi:10.1371/journal.pone.0025606)

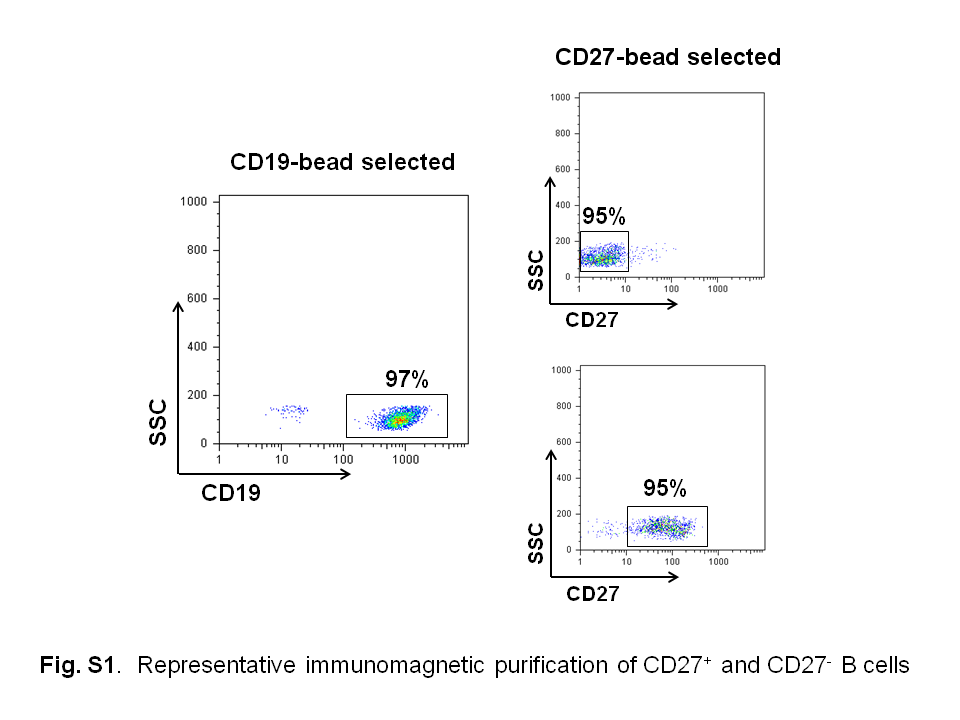

Supplement: Figure S1 — Representative immunomagnetic purification of CD27+ and CD27− B cells. (TIF) [file pone.0025606.s001.tif]
